# Prevalence Estimates for Pharmacological Neuroenhancement in Austrian University Students: Its Relation to Health-Related Risk Attitude and the Framing Effect of Caffeine Tablets

**DOI:** 10.3389/fphar.2018.00494

**Published:** 2018-06-12

**Authors:** Pavel Dietz, Benedikt Iberl, Emanuel Schuett, Mireille van Poppel, Rolf Ulrich, Matteo Christian Sattler

**Affiliations:** ^1^Research Group of Physical Activity and Public Health, Institute of Sports Science, University of Graz, Graz, Austria; ^2^Working Group Social and Health Sciences of Sport, Institute for Sports and Sports Science, Karlsruhe Institute of Technology, Karlsruhe, Germany; ^3^Research Group of Cognition and Perception, Institute of Psychology, University of Tübingen, Tübingen, Germany

**Keywords:** message frame, cognitive bias, cognitive enhancing drugs, risk behavior, substance abuse detection, statistical distributions, epidemiologic methods, randomized response technique

## Abstract

**Background:** Pharmacological neuroenhancement (PN) is defined as the use of illicit or prescription drugs by healthy individuals for cognitive-enhancing purposes. The present study aimed (i) to investigate whether including caffeine tablets in the definition of PN within a questionnaire increases the PN prevalence estimate (framing effect), (ii) to investigate whether the health-related risk attitude is increased in students who use PN.

**Materials and methods:** Two versions of a paper-and-pencil questionnaire (first version included caffeine tablets in the definition of PN, the second excluded caffeine tablets) were distributed among university students at the University of Graz, Austria. The unrelated question model (UQM) was used to estimate the 12-month PN prevalence and the German version of the 30-item Domain-Specific Risk-Taking (DOSPERT) scale to assess the health-related risk attitude. Moreover, large-sample *z*-tests (α = 0.05) were performed for comparing the PN prevalence estimates of two groups.

**Results:** Two thousand four hundred and eighty-nine questionnaires were distributed and 2,284 (91.8%) questionnaires were included in analysis. The overall PN prevalence estimate for all students was 11.9%. One-tailed large-sample *z*-tests revealed that the PN estimate for students with higher health-related risk attitude was significantly higher compared to students with lower health-related risk attitude (15.6 vs. 8.5%; *z* = 2.65, *p* = 0.004). Furthermore, when caffeine tablets were included into the example of PN, the prevalence estimate of PN was significantly higher compared to the version without caffeine tablets (14.9 vs. 9.0%; *z* = 2.20, *p* = 0.014).

**Discussion:** This study revealed that the PN prevalence estimate increases when caffeine tablets are included in the definition of PN. Therefore, future studies investigating the prevalence of, and predictors for, PN should be performed and interpreted with respect to potential framing effects. This study further revealed that the PN prevalence estimate is increased in students with a higher health-related risk attitude compared to students with a lower one. Therefore, future education and prevention programs addressing PN in the collective of students should not only inform about potential side effects of its use but also address the limited effects on cognition and potential alternatives of PN.

## Introduction

The term “pharmacological neuroenhancement (PN)” – also called “pharmacological cognitive enhancement” – is generally defined as the use of illicit (e.g., illicit stimulants, cocaine, ecstasy) or prescription drugs (e.g., stimulants such as methylphenidate and amphetamines, modafinil as well as antidementives and antidepressants) by healthy individuals for cognitive-enhancing purposes such as improving vigilance, attention, concentration, or mood (Franke and Lieb, 2010; Dietz et al., 2013b; Sattler, 2016). In the last decade, a considerable number of epidemiological studies using different survey methods investigated the prevalence of PN in various populations. For example, based on a survey by a large German health insurance company, a lifetime prevalence of 5% for PN was reported for the general working population in Germany (DAK, 2009), and lifetime prevalences of about 20% for scientists (Maher, 2008), surgeons (Franke et al., 2013), and economists (Dietz et al., 2016b). In addition, a systematic review by Wilens et al. (2008) estimated a lifetime prevalence for the use of stimulants including prescription and illicit drugs of 5–9% in graduate and high school students, and 5–35% among college students in the United States (Wilens et al., 2008). Similar results were obtained for student collectives in Western Europe. For example, lifetime prevalences for the use of prescription drugs of 8% and 9% were reported for pupils in Germany (Wolff and Brand, 2013) and Switzerland (Liakoni et al., 2015), respectively. Moreover, for university students the lifetime prevalence was about 5% for the use of prescription drugs for cognitive-enhancing purposes among German students (Sattler and Wiegel, 2013), 7.6% among Swiss students (Maier et al., 2013), around 2% among Dutch students (Schelle et al., 2015), 16% for the use of drugs among Italian students (Castaldi et al., 2012), and about 4% for the use of methylphenidate, 6% for modafinil, and 2% for Adderall for cognitive-enhancing purposes among students in Ireland/United Kingdom (Singh et al., 2014). Using an indirect survey technique, Dietz et al. (2013a) estimated a 12-month prevalence of 20% for PN for German university students including prescription drugs, illicit drugs, and caffeine tablets.

The heterogeneity of the prevalence estimates across studies can be attributed to various factors. For example, some studies employed direct and others indirect questioning methods (Lensvelt-Mulders et al., 2005b; Simon et al., 2006; Franke et al., 2013) when assessing such sensitive issues (Sieber and Stanley, 1988; Lee and Renzetti, 1990). In addition, some studies assessed the prevalence for a single substance (e.g., methylphenidate only) while other studies for a whole group of substances. Finally, studies also differed with respect to the definition and description of PN (Sattler, 2016; Schleim and Quednow, 2017). The latter issue can produce framing effects that biases people’s responses (Rothman et al., 1993; Stocké, 2002; Plous, 2007). In order to omit such bias effects like social desirability, Dietz et al. (2013a,b) conducted two surveys to estimate PN prevalence in university students and triathletes using an indirect survey technique. In these latter studies, the terms “illicit and prescription drugs” were explicitly defined in the survey questionnaire as “…substances which can only be prescribed by a doctor, are available in a pharmacy, or can be bought on the black market (e.g., caffeine tablets, stimulants, cocaine, methylphenidate, modafinil, beta-blockers) and are used to enhance your cognitive performance.” Although caffeine tablets are not illicit and hence a prescription is not needed to receive them, the authors included caffeine tablets in their example of PN because in Germany (in contrast to the United States), caffeine tablets can only be bought in pharmacies and not in supermarkets or drug-stores. In addition, the consumption of caffeine tablets differs markedly from the consumption of a cup of coffee, because coffee may also be consumed for appetite whereas the only reason for consuming caffeine tablets would be to reduce fatigue (Franke et al., 2011b, 2015). Other authors have argued that prevalence estimates for PN of 20% in university students and 15.1% in triathletes are particularly a consequence of this inclusion of caffeine tablets in the definition of PN (Maier et al., 2013; Liakoni et al., 2015; Maier and Schaub, 2015; Schleim and Quednow, 2017), even though other studies revealed quite comparable results without including caffeine tablets (Maher, 2008; Franke et al., 2013; Dietz et al., 2016b). Nonetheless, although postulated by scientists to be needed (Schleim and Quednow, 2017), no systematic research has been conducted whether the inclusion of caffeine tablets in the definition of PN increases PN prevalence estimates. Given that 11% of students (Franke et al., 2011b) and 13% of surgeons (Franke et al., 2015) reported to have used caffeine tablets for cognitive-enhancing purposes once in their life, we hypothesized that including caffeine tablets in the definition of PN would increase PN prevalence estimates compared to excluding caffeine tablets in the definition.

Furthermore, PN consumption is, depending on dosage, associated with diverse adverse acute and chronic effects on physical and mental health, has been assumed to lead to addiction and produces a gateway to other drugs (Kumar, 2008; Dietz et al., 2013b; Wolff and Brand, 2013; LaBotz and Griesemer, 2016). For example, stimulant use is associated with the risk of cardiovascular events, hypertonia, tachycardia, and even sudden cardiac death (Kumar, 2008). In addition, long-term use of methylphenidate has been associated with neuronal changes comparable to those of cocaine use (Steiner and van Waes, 2013; Noble et al., 2015). A qualitative study among 19 university students in Australia revealed that students were aware of potential side effects of PN (Partridge et al., 2013). Fortunately, knowing potential side effects of PN reduces the willingness to consume PN (Sattler et al., 2014). Consequently, it has been assumed that the prospect of expected side effects from drug use acts as a protective factor against PN (Sattler and Wiegel, 2013). However, knowing about health risks does not necessarily prevent everybody from using PN, and several studies indicated that increased risk-taking behavior is associated with drug use (Khan et al., 2012; Noble et al., 2015; Nicholls et al., 2017). Furthermore, a study performed by Maier et al. (2015) comparing personality traits/attitudes of users of PN and non-users indicated that risk-taking-related personality traits/attitudes such as novelty seeking, self-reported impulsivity as well as antisocial personality were increased in users of PN compared to controls (Maier et al., 2015). Therefore, we hypothesized that people who consume PN have an increased health-related risk attitude compared to people who do not consume PN.

The present study aimed to address the following: (i) to investigate whether including caffeine tablets in the definition of PN within a questionnaire increases the PN prevalence estimate, (ii) to investigate whether the health-related risk attitude is increased in students who use PN, by providing first evidence on the prevalence of PN in Austrian university students. Investigating these knowledge gaps is of public health concern in order to develop more individually tailored education and prevention concepts.

## Materials and Methods

### Survey Procedure

A paper-and-pencil survey was conducted among university students at the University of Graz, Austria. The study administration online platform of the university was used to identify all major classes from different disciplines during the summer term of 2017. Two weeks before the survey was distributed, all teachers/lecturers of the identified classes were informed about the study and the procedure by email. The email also requested consent to distribute the survey in their classes. When consent was obtained, a trained team of assistants visited the classes one-time and distributed the questionnaires (two versions, one per participant; for more details see the section on “questionnaire” below). In a short verbal introduction, the assistants stressed the anonymity of the study and told the students to fill in the questionnaire immediately and to drop it into black boxes that were set up in the lecture hall. They further emphasized that all questionnaires had to be returned regardless if they were completed or not in order enable an accurate calculation of the response rate. The whole procedure lasted 10–15 min. Ethical approval to conduct this study was obtained by the Ethics Committee of the University of Graz, Austria (GZ. 39/40/63 ex 2016/17).

### Questionnaire

The anonymous paper-and-pencil questionnaire was two pages long and, after a short introduction explaining the content of the study and that participation would be anonymous and voluntary, was comprised of three parts. The *first* part addressed the 12-month prevalence of PN using the unrelated question model (UQM; Greenberg et al., 1969), a randomized response technique developed specifically to obtain more valid prevalence estimates when sensitive topics are studied by guaranteeing anonymity (Warner, 1965, 1971). The methodological background of the UQM as used for the present study has been explicitly described in several previous articles (Dietz et al., 2013a,b, 2016a; Franke et al., 2013, 2017; Schröter et al., 2016) and will therefore not be repeated in detail. In short, participants had to consider a certain birthday (of their mother). If this birthday was in the first third of the month (1st to 10th day), they had to answer a neutral question and if not, they had to answer the sensitive question regarding the prevalence of PN. Thus, the probability for receiving the neutral question was 120/365.25 and the probability for receiving the sensitive question was 245.25/365.25. The neutral question asked whether the birthday they considered is in the first half of the year (before 1st of July). Therefore, the probability for answering the neutral question with ‘yes’ (denoted as *π_n_*) was 181.25/365.25. The proportion of ‘yes’ responses with respect to the sensitive question (i.e., the prevalence estimate 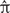
_s_) can then be derived from the proportion of total ‘yes’ responses in the sample (denoted as *a*). A specific arithmetic example is given in the publication of Franke et al. (2013).

The translated wording of the sensitive question regarding PN was “for the purpose of enhancing your cognitive performance during studying, have you used substances which are only available in a pharmacy, can be prescribed by a doctor or can be bought on the black marked during the last 12 month (examples).” We used two different questionnaire versions containing different versions of the example for substances. The first version included caffeine tablets in the example “*(e.g., caffeine tablets, stimulants, cocaine, methylphenidate, beta-blockers, modafinil)*” and the second did not “*(e.g., stimulants, cocaine, methylphenidate, beta-blockers, modafinil).*” This procedure enabled us to calculate two different PN prevalence estimates, one for including caffeine tablets and one for excluding caffeine tablets. The order for distributing the two questionnaire versions was determined by simple randomization (Altman and Bland, 1999; Schulz and Grimes, 2007) using Windows Excel (questionnaires with an uneven number included caffeine tablets, questionnaires with an even numbers did not). Within the *second* part of the questionnaire, we assessed the health-related risk attitude using the German version of the 30-item Domain-Specific Risk-Taking (DOSPERT) scale for adults (Blais and Weber, 2006). The DOSPERT scale contains 30 items in five distinct domains of risk taking (six items per domain): ethical, financial, health/safety, recreational, and social. The six items for health/safety were adapted to our questionnaire in order to obtain the domain specific score. On a seven-point Likert scale (1 = very unlikely, 2 = unlikely, 3 = somewhat unlikely, 4 = unsure, 5 = somewhat likely, 6 = likely, 7 = very likely) the following items for the health/safety domain had to be rated: how likely is it for you (i) drinking five or more glasses of alcohol at one evening, (ii) engaging in unprotected sex, (iii) driving a car without wearing a seat belt, (iv) riding a motorcycle without a helmet, (v) sunbathing without sunscreen, and (vi) walking home alone at night in an unsafe area of town. For each student, the average rating to these items was calculated. This score was used to classify the participants into groups of higher or lower health-related risk attitude and enabled us to calculate separate PN prevalence estimates for these two subgroups. Finally, the *third* part of the questionnaire contained four items concerning the participant’s characteristics: semester (continuous), field of study (nominal), age (continuous), and gender (male/female). The complete questionnaire is provided in the Supplementary Material to this article.

### Statistics

Descriptive data are presented as mean ± SD values for continuous scaled variables and as numbers and percentages for non-continuous scaled variables using SPSS software, version 22. Prevalence estimates (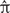
_s_) for PN are presented as percentages with 95% confidence intervals (CI) and standard error (SE) using Matlab version R2015a. Moreover, R software version 3.2.3 was used to perform large-sample *z*-tests for comparing the prevalence estimates of PN between two groups (for example between participants with lower and higher health-related risk attitude). The central hypotheses of the study regarding the influence of caffeine tablets and health-related risk attitude on PN prevalence estimate were directed hypotheses and analyzed one-tailed (right-tailed, α = 0.05). Non-directed variables (gender, age, and semester) were analyzed two-tailed (α = 0.05). Therefore, the continuous variables ‘age’ and ‘health related risk attitude’ were dichotomized by median and in a second analysis the variable ‘semester’ manually by visual splitting.

## Results

A total number of 2,489 questionnaires were distributed among students from the University of Graz and 2,464 (99%) were returned. One hundred and eighty students stated that they had already participated in the survey in a previous class/lecture and hence these students were excluded from data analysis resulting in a total number of 2,284 (91.8%) questionnaires for the analysis. Mean age was 22.4 years (*SD* = 5.2 years), mean semester was 3.7 (*SD* = 2.5), 1,343 (59.1%) participants were female, and 1,138 (49.8%) obtained the questionnaire version that included caffeine tablets in the example of PN. The 16 different fields of study stated by the respondents were summarized into five groups taking the local faculty affiliation of the different fields as well as previous classification schemes (Dietz et al., 2013a) into account. The mean health-related risk attitude assessed by the DOSPERT scale was 22.3 (*SD* = 5.9). The respondent’s characteristics are presented in **Table 1**.

**Table 1:** Characteristics of the respondents.

Variable	Value
**Gender**, no (%)	
Female	1,343 (59.1)
Male	928 (40.9)
**Age**, range, years (mean ± SD)	16–85 (22.4 ± 5.2)
**Semester**, range (mean ± SD)	1–22 (3.7 ± 2.5)
**Field of study^#^**, no (%)	
Technical studies and informatics	216 (9.8)
Natural sciences	431 (19.5)
Humanities, social sciences, languages and sport	597 (27.0)
Economy, law and USW	484 (21.9)
Medicine, pharmacy and psychology	486 (22.0)
**Health-related risk attitude**, range (mean ± SD)	6–42 (22.3 ± 5.9)
**Questionnaire version**, no (%)	
With caffeine tablets	1,138 (49.8)
Without caffeine tablets	1,146 (50.2)

*USW, Umweltsystemwissenschaften (environmental systems sciences). ^#^The items for the variable field of study were grouped on the basis of two previous studies and modified according to the local affiliation of the different fields to the faculties.*

**FIGURE 1:**
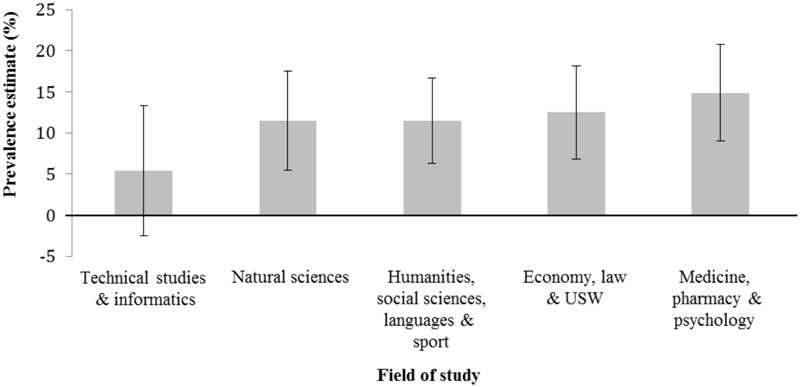
Estimated 12-month prevalence and 95% confidence intervals for pharmacological neuroenhancement separated for field of study. USW, Umweltsystemwissenschaften (environmental systems sciences).

The overall PN prevalence estimate for all students was 11.9% (**Table 2**). One-tailed large-sample *z*-tests revealed that PN estimate for students with higher health-related risk attitude was significantly higher (15.6%) compared to the group of students with a lower (8.5%) health-related risk attitude (*z* = 2.65, *p* = 0.004). Furthermore, when caffeine tablets were included into the example of PN, the prevalence estimate of PN was significantly higher (14.9%) compared to the version without caffeine tablets (9.0%, *z* = 2.20, *p* = 0.014). Two-tailed large-sample *z*-tests revealed that prevalence estimates were not significantly different between female (10.2%) and male (14.9%) participants (*z* = 1.70, *p* = 0.088), participants aged younger than or equal to 21 years (12.3%) and older (11.7%) participants (*z* = 0.22, *p* = 0.826) as well as between students studying in the first (12.5%) or higher than first (11.5%) year (*z* = 0.37, *p* = 0.712). **Figure 1** presents the prevalence estimates together with their 95% confidence intervals for the five different fields of study indicating that the prevalence estimate is the lowest in technical studies and informatics (5.4%) and the highest in medicine, pharmacy, and psychology (14.9%). The prevalence estimates for the remaining three groups were 11.5%, 11.5%, and 12.5%, respectively.

**Table 2:** Estimated 12-month prevalence for pharmacological neuroenhancement.

Variable	‘Yes’	‘No’	*a*	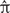 _s_(%)	*SE* ( 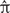 _s_)	95% CI
**All students** (*n* = 2,275)^∗^	553	1,722	0.243	11.9	1.3	9.3–14.5
**Gender**						
Female	310	1,029	0.232	10.2	1.7	6.8–13.6
Male	243	680	0.263	14.9	2.2	10.7–19.2
**Age^#^**						
≤21 years	329	1,012	0.245	12.3	1.8	8.8–15.7
>21 years	222	698	0.241	11.7	2.1	7.5–15.8
**Semester**						
1st or 2nd (first year)	295	899	0.247	12.5	1.9	8.9–16.2
>2nd	249	787	0.24	11.5	2.0	7.6–15.4
**Health-related risk attitude^#^**						
≤22	256	908	0.22	8.5	1.8	4.9–12.0
>22	296	810	0.268	15.6	2.0	11.7–19.5
**Questionnaire version**						
With caffeine tablets	298	835	0.263	14.9	2.0	11.1–18.7
Without caffeine tablets	255	887	0.223	9.0	1.8	5.4–12.6

*^∗^Of the 2,284 students that filled in the questionnaire, nine students provided invalid results on the RRT question resulting in a total number of 2,275 valid responses. In detail, three students made no cross and six students made two crosses by answering the RRT question but only one cross (‘yes’ OR ‘no’) is allowed. ^#^Dichotomized by median.*

## Discussion

The present study addressed two questions. First, whether the PN prevalence estimate increases when caffeine tablets are included in the definition of PN. Second, whether the PN prevalence estimate increases with increasing health-related risk attitude. Therefore, a randomized-response survey was conducted among university students at the University of Graz. Regarding the first question, the present results support the assumption (Maier et al., 2013; Liakoni et al., 2015; Maier and Schaub, 2015; Schleim and Quednow, 2017) that the questionnaire version including caffeine tablets in the PN definition increased the PN prevalence estimate (14.9% without vs. 9.0% with caffeine tablets). This difference of about 6% is consistent with the last-year prevalence for the use of caffeine tablets for cognitive-enhancing purposes, which was also reported to be about 6% (Franke et al., 2015). The results also demonstrate the relevance of caffeine tablets from a public health point of view, since the consumption of high dosages of caffeine is discussed to be associated with comparable adverse health effects as described for prescription and illicit psychoactive drugs (Jackson et al., 2013; Temple et al., 2017). In addition, according to several studies, high concentrations of caffeine (of 80 mg/L) are considered lethal (Banerjee et al., 2014; Silva et al., 2014). However, from a methodological point of view we have to stress that participants who responded to version 2 of the questionnaire (the version without caffeine tablets) may still have considered caffeine tablets as PN. Consequently, we cannot directly assess the prevalence of caffeine tablets but only calculate the difference of prevalence estimation of PN between the two questionnaire versions (with and without caffeine tablets).

Regarding the health-related risk attitude, the present results provide evidence that the PN prevalence is higher in participants with a higher health-related risk attitude (15.6%) compared to participants with a lower health-related risk attitude (8.5%). Consequently, our study supports the results of a previous study performed among university students in Switzerland (Maier et al., 2015). In their study, risk-taking-related personality traits/attitudes such as novelty seeking, self-reported impulsivity as well as antisocial personality were increased in users of PN compared to controls. Likewise, previous studies reported comparable associations between risk attitude and the use of other substances such as alcohol, tobacco, illicit drugs, and doping substances (Khan et al., 2012; Noble et al., 2015; Nicholls et al., 2017). This association might be of particular interest for the student population, since the Centers for Disease Control and Prevention of the United States and others have shown that students engage in various health risk behaviors and ignore preventive safety habits, which may have long-term implications for their health (Centers for Disease Control and Prevention, 1997; Steptoe and Wardle, 2001; Steptoe et al., 2002; Von Ah et al., 2004; Kann et al., 2013). Therefore, we conclude that prevention and education programs addressing PN in the collective of students likely fail if they only inform about potential side effects of its use (approach of deterrence). In fact, such programs have to further share knowledge about the limited efficacy of PN in healthy individuals (Repantis et al., 2010; Ullrich et al., 2015) and that using these substances is not associated with better school marks (Franke et al., 2011a) nor with better academic performance (McCabe et al., 2005). In addition, students have to be informed about potential alternatives of PN which are discussed to have cognitive-enhancing effects, such as sports and exercise (Tomporowski, 2003; Lambourne and Tomporowski, 2010; Dietz, 2013), nutrition (Chung et al., 2012), sleep (Diekelmann and Born, 2010), or meditation (Lutz et al., 2008; Sedlmeier et al., 2012).

The overall 12-month PN prevalence estimate of 11.9% for the collective of university students in Graz, Austria is quite in the middle of the heterogeneous prevalences reported for university students from other countries in Europe (e.g., prevalence of 2% since the start of their university studies for Dutch (Schelle et al., 2015) vs. 12-month prevalence of 20% for German (Dietz et al., 2013a) university students. As stated above, the differences across studies may be attributed to various methodological factors. Additionally, the Dutch study did not include caffeine tablets whereas the German study did. Since the present overall prevalence estimate is a mix of both versions (with caffeine tablets and without) it is quite plausible that this PN prevalence estimate is quite in the middle of the Dutch and the German prevalence. Finally, although the present study was the first large survey assessing the prevalence of PN in Austrian University students using RRT, the present results are not representative for Austria because the survey was restricted to the University of Graz. Nonetheless, we were able to demonstrate that the used study design and survey techniques are highly appropriate to detect cross-sectional associations between potential predictor variables and PN.

The variables gender and semester did not significantly influence PN prevalence estimates. For gender, our results support the outcomes of other studies indicating that there is no difference in the PN usage between female and male students (Teter et al., 2006; Franke et al., 2011a; Mache et al., 2012). Yet some studies reported gender differences, with males having a higher risk for PN compared to females (DeSantis et al., 2008; Arria et al., 2011). In a recent meta-analysis by Benson et al. (2015), of 19 studies on gender differences in misuse of stimulant medication among students, 13 studies reported a higher prevalence for males compared to females, whereas six studies observed no difference. This heterogeneous result pattern may reflect differences in methodology, for example, by using cross-sectional and longitudinal study designs, different survey techniques as well as assessing prevalences for different time periods such as lifetime versus last-year prevalence (Benson et al., 2015). In contrast to the present study, a survey among university students in Germany reported that students of the first semester were of higher risk for PN compared to higher semester students. In the present survey, only 105 participants were first semester students. Therefore, we could not calculate a reliable PN prevalence estimate for the first semester students (Ulrich et al., 2012). To examine the influence of time spent at the university on the prevalence of PN, future studies should collect longitudinal data, which may enable researchers to assess changes in PN consumption over time.

One limitation of the present study might be that we used a self-designed question to assess the overall prevalence of PN. This has been done for the following reason. It is known from previous studies (e.g., doping in athletes) that non-professional people are not aware about which substances are illicit or need to be prescribed by a doctor. Therefore, we provided a simple explanation by what we mean with “for the purpose of enhancing your cognitive performance during studying, have you used substances which are only available in a pharmacy, can be prescribed by a doctor or can be bought on the black marked during the last 12 month (examples)” (Dietz et al., 2013a,b). One objection against this definition might be that it excludes substances that can only be bought in a pharmacy (e.g., high dosages of vitamins). Therefore, we provided concrete examples of substances in order to increase the comprehension of our question. It is likely that prevalence estimates are influenced by how such questions are posed. For example, the expression “prescription drugs for cognitive-enhancement” seems ambivalent for readers and hence may produce prevalence estimates that are too small compared to the true prevalence. Another limitation of the study might be that we were not able to identify specific substances for PN by using the present RRT design to investigate an overall prevalence estimate of PN. This gap should be closed by future RRT studies that are tailored to address specific substance prevalences.

Unrelated question model was used in the present study to assess the PN prevalence estimate. UQM is one technique among several RRTs [e.g., the *forced response method*, the *item count technique*, the *crosswise method*, the *cheater detection model* (CDM)]. In their meta-analysis of 38 randomized response validation studies, Lensvelt-Mulders et al. (2005a) have concluded that although RRT results are more valid compared to results of conventional survey techniques, there is still room for improvement (Lensvelt-Mulders et al., 2005a). Using the UQM participants get randomly assigned to answer one of two questions, the sensitive or a neutral one. However, a participant that has been assigned to answer the sensitive answer may avoid this question and respond to the neutral question instead. As shown by Ulrich et al. (2018, Supplementary Material, Section 4.3), this non-compliance does not meaningfully distort the estimate obtained under the standard UQM assumptions. Moreover, a previous study has shown that UQM and CDM, a model taking potential cheating of participants into account, delivered comparable prevalence estimates for a sensitive item (Schröter et al., 2016).

## Conclusion

This study revealed that the PN prevalence estimate increases when caffeine tablets are included in the definition of PN. Therefore, future studies investigating the prevalence of, and predictors for, PN should be performed and interpreted with respect to potential framing effects. This study further revealed that the PN prevalence estimate is increased in students with a higher health-related risk attitude compared to students with a lower one. Therefore, future education and prevention programs addressing PN in the collective of students should not only inform about potential side effects of its use but also address the limited effects on cognition and potential alternatives of PN.

## Author Contributions

PD, BI, ES, RU, and MS made substantial contributions to the conception and design of the study, the data acquisition, analysis, interpretation of data, and the preparation of the manuscript. MvP made substantial contributions to the interpretation of data and the preparation of the manuscript. All authors proofread and accepted the final version of the manuscript.

## Conflict of Interest Statement

The authors declare that the research was conducted in the absence of any commercial or financial relationships that could be construed as a potential conflict of interest. The reviewer JD and handling Editor declared their shared affiliation.
