# Supplementary material for: Prevalence Estimates for Pharmacological Neuroenhancement in Austrian University Students: Its Relation to Health-Related Risk Attitude and the Framing Effect of Caffeine Tablets
Source: Front Pharmacol. 2018 Jun 12;9:494. doi: 10.3389/fphar.2018.00494 (PMC6006370; doi:10.3389/fphar.2018.00494)
Supplement: Supplementary file 1 [file Data_Sheet_1.PDF]

EBERHARD KARLS UNIVERSITÄT TÜBINGEN

KARL-FRANZENS-UNIVERSITÄT GRAZ
UNIVERSITY OF GRAZ
Institute of Sport Science

Fragebogen

Prof. R. Ulrich (Psychologie, Eberhard Karls Universität Tübingen),
Dr. P. Dietz und M. Sattler (Sportwissenschaft, Karl-Franzens-Universität Graz)

Liebe Teilnehmerinnen und Teilnehmer,

wir führen eine Studie durch, um den Gebrauch von Substanzen zu untersuchen, die die geistige Leistungsfähigkeit im Rahmen des Studiums verbessern. Der Fragebogen ist so gestaltet, dass Ihre Antworten vollkommen anonym sind. Wie Sie sehen werden, können wir aufgrund Ihrer Antwort nicht entscheiden, ob Sie eine solche Substanz zu sich genommen haben oder nicht. Erst wenn wir von mehreren hundert Teilnehmern die Antworten erhalten haben, können wir aufgrund der Durchschnittswerte aller Teilnehmer bestimmen, wie viele von Ihnen die heiklen Fragen in diesem Fragebogen bejaht haben. Die Teilnahme an dieser Studie ist freiwillig. Falls Sie nicht teilnehmen wollen, so werfen Sie diesen Fragebogen unausgefüllt in die dafür vorgesehene Box. Der Fragebogen besteht aus drei Teilen. Bitte lesen Sie die Fragen in jedem Teil genau durch und beantworten Sie diese ehrlich.

Teil 1

Bei den folgenden Fragen zur Substanzeinnahme mit dem Ziel der Steigerung der geistigen Leistungsfähigkeit im Rahmen des Studiums (Wachheit, Aufmerksamkeit, Konzentration) möchten wir Ihnen absolute Anonymität gewährleisten. D.h. selbst wenn Ihnen beim Ankreuzen jemand direkt zuschaut, oder selbst wenn Sie jemandem diesen Fragebogen persönlich zeigen, wird diese Person nicht nachvollziehen können, auf welche Frage Sie unten mit Ja oder Nein geantwortet haben.

Denken Sie bitte an den <u>Geburtstag Ihrer Mutter</u>.
Liegt dieser Geburtstag im ersten Drittel des entsprechenden Monats (1. bis 10. Tag)? Wenn ja, dann beantworten Sie bitte wahrheitsgemäß Frage A, ansonsten bitte wahrheitsgemäß Frage B.

Frage A: Liegt der Geburtstag Ihrer Mutter in der ersten Jahreshälfte, also vor dem 1. Juli eines Jahres?

Frage B: Haben Sie zur Steigerung Ihrer **geistigen Leistungsfähigkeit** im Rahmen des Studiums in den letzten 12 Monaten Substanzen eingenommen, die es nur in der Apotheke, beim Arzt oder auf dem Schwarzmarkt gibt (z.B. Koffeintabletten, Aufputschmittel, Kokain, Methylphenidat/Ritalin®, Beta-Blocker, Modafinil)?

Ihre Antwort auf **Frage A <u>oder</u> auf Frage B** (nur Sie wissen, worauf Sie hier antworten) lautet:

Ja ☐ **Nein** ☐

Teil 2

Nun möchten wir noch etwas über Ihr Risikoverhalten im Allgemeinen erfahren.

Geben Sie für jede der folgenden Aussagen an, mit welcher **Wahrscheinlichkeit** Sie der genannten Aktivität oder Verhaltensweise nachgehen würden. Benutzen Sie dafür bitte folgende Skala von **1 bis 7** und tragen Sie die Zahl in das dafür vorhergesehene Kästchen ein.

1	2	3	4	5	6	7
sehr unwahrscheinlich	unwahrscheinlich	eher unwahrscheinlich	nicht sicher	eher wahrscheinlich	wahrscheinlich	sehr wahrscheinlich

1) ...fünf oder mehr Gläser Alkohol an einem einzigen Abend zu sich nehmen? ☐

2) ...sich auf ungeschützten Sex einlassen? ☐

3) ...sich im Auto nicht anschnallen? ☐

4) ...ohne Helm Fahrrad fahren? ☐

5) ...sich einem Sonnenbad aussetzen, ohne sich eingecremt zu haben? ☐

6) ...nachts alleine durch einen unsicheren Stadtteil nach Hause gehen? ☐

Teil 3

Zuletzt benötigen wir noch folgende Angaben von Ihnen:

1)	Fachsemester: ☐	2)	Studiengang/ -gänge:	☐
3)	Alter in Jahren: ☐	4)	Geschlecht:	☐ Männlich ☐ Weiblich

Wir bedanken uns für Ihre Teilnahme!
